# Using a sensitive luciferase immunoprecipitation system (LIPS) to detect HTLV-I/II seroindeterminates in Jamaica

**DOI:** 10.1186/1742-4690-8-S1-A249

**Published:** 2011-06-06

**Authors:** Yoshimi Akahata, Anna Abrams, Elizabeth Maloney, Matthew McCormick, Steven Jacobson

**Affiliations:** 1 Neuroimmunology Branch, National Institute of Neurological Diseases and Stroke, National Institutes of Health, Bethesda, MD, USA; 2 Division of Epidemiology, Food and Drug Administration, Silver Spring, MD, USA

## 

Human T-cell Leukemia Virus type I (HTLV-I) infects an estimated 15-20 million persons worldwide. A number of diseases have been associated with this virus including adult T-cell leukemia/lymphoma (ATLL), HTLV-associated myelopathy/tropical spastic paraparesis (HAM/TSP), HLTV-I uveitis, and HTLV-I–associated infective dermatitis. The screening process for HTLV-I among blood banks includes detection by an enzyme immunoassay (EIA) followed by a confirmatory western blot (WB). Seropositive results are defined by the presence of all core bands (p19, p24, and p53) as well as the band specific for recombinant HTLV-I Env glycoprotein. However, numerous cases have been documented in which a positive response was detected by ELISA, but WB displayed an incomplete banding pattern. These samples were then categorized as seroindeterminates. Using the LIPS assay, 60% of the 53 seroindeterminate samples previously screened by ELISA and WB displayed anti-HTLV-I Gag, Env or Tax antibody responses (based on 2 standard deviations above the mean value for the HTLV-I negative group). The LIPS assay was also able to detect anti-HTLV-I antibody responses in 6% of the 167 samples determined to be HTLV-I ELISA negative. A number of these samples were subsequently found to have an indeterminate WB pattern. Although the significance of these HTLV-I/II seroindeterminates is unclear, it may suggest a much higher prevalence of exposure to HTLV-I/II than previously estimated, as seroindeterminate samples may indicate exposure to the virus. The LIPS assay is a sensitive and high throughput antibody detection assay which may prove to be a useful tool in HTLV-I related clinical investigations.

